# Cryoablation as an organ-preserving therapy for low rectal cancer a comprehensive protocol and institutional perspective

**DOI:** 10.3389/fonc.2026.1757590

**Published:** 2026-07-16

**Authors:** Xuejun Jiang, Feng Zhou, Zujin Ji, Xinyi Lei, Fangjun Yuan

**Affiliations:** 1 Department of Colorectal and Anal Surgery, Sinopharm Dongfeng General Hospital, Hubei University of Medicine, Shiyan, Hubei, China; 2 Department of General Surgery, Sinopharm Dongfeng General Hospital, Hubei University of Medicine, Shiyan, Hubei, China

**Keywords:** cryosurgery, immuneactivation, intervention treatment, rectal neoplasm, sphincter preservation

## Abstract

Cryoablation is an interventional approach which induces localized tumor destruction. It is used to treat patients with low rectal cancer (LRC) to preserve their sphincters and avoid permanent colostomies. This article provides a systematic illustration of cryoablation for LRC, encompassing its core technical principles, indications and contraindications. Furthermore, it elaborates on pre-procedural preparation, standardized operative steps, post-cryoablation management, potential complications and mitigation and long-term follow-up protocols. Rooted in five decades of institutional experience, we seek to standardize the procedure and establish it as a credible, evidence-based alternative approach for well-selected patients, especially those who are not candidates for or who decline radical resection.

## Introduction

Cryoablation is a minimally invasive interventional technique that achieves localized tumor ablation via precise placement of a cryoprobe at the target lesion. Inside the cryoprobe, circulating refrigerants undergo phase changes to absorb heat, which rapidly lowers the lesion’s temperature to below -40 °C and thus induces cellular destruction (1). Although first conceptualized in the 19th century, modern cryoablation achieves tumor destruction through multiple synergistic mechanisms: physical cellular damage, hypothermia-induced cellular necrosis or apoptosis, impairment of tumor vasculature (resulting in ischemia), and induction of antitumor immune responses (2).These mechanisms are characterized by both direct cellular injury and activation of systemic immune responses, distinguishing cryoablation from other ablative modalities.

Currently, cryoablation is widely used in the treatment of malignancies, including lung tumors and prostate cancer (3–9). However, its role in the management of LRC remains less established compared to other modalities, despite the ongoing need for sphincter-preserving treatments. Although advances in sphincter-preserving techniques have improved functional outcomes in some LRC patients (10, 11), clinical gaps persist—especially in patients who refuse radical surgery or have comorbidities that prevent extensive surgical intervention. The core goal of colorectal surgeons remains balancing oncologic radicality and functional preservation (12, 13).

Within this clinical practice, our institution has employed cryoablation as a specialized, sphincter-preserving option for select LRC patients since 1976 (14). In this article, we aim to address this gap by presenting a comprehensive, standardized clinical protocol and sharing the key insights from our 50-year experience, which include the procedure’s safety, feasibility and unique role in organ-preservation era for LRC.

## Rectal cryoablation: feasibility and advantages

The lower rectum is encased by the rectal proper fascia—a dense connective tissue layer that limits ice ball spread and mitigates perforation risk. Additional reported benefits include simplified anesthesia, high reproducibility and a short learning curve which enhance its clinical utility (15–18).This anatomical feature establishes cryoablation as a feasible option for selected LRC patients.

### Ethical considerations and data management

This study has been approved by the Ethics Committee of the Sinopharm Dongfeng General Hospital (IRB: LC-2022-001).This research adheres to the latest iteration of the Declaration of Helsinki and complies with Chinese laws and regulations.

### Cryoablation devices

Our institution used the APCA1–3 system for rectal cryoablation, due to its accelerated cooling rate (which reduced procedural time) and improved ice ball precision (which minimized injury to adjacent anatomical structures)-a particularly important feature when ablating lesions on the anterior rectal wall due to the proximity to critical structures. Other commercial cryosystems for tumor cryoablation have been reported in the literature (19, 20).

### Cryoprobe application methods

Cryoprobe placement varies by probe design, with three primary approaches (**Table 1**). For LRC, only contact cryotherapy (pie-shaped probe) is indicated, as it enables superficial, targeted ablation of lesions within 5 cm of the anal verge—avoiding deep tissue injury to the rectal wall or surrounding organs (21–27).

**Table 1:** Probe type selection for LRC.

Probe type	Application scenario	LRC relevance
Pie-shaped (contact)	Small, superficial solid tumors (≤3 cm diameter)	Direct surface contact ensures full tumor coverage
Needle-type (insertion)	Deep/large solid tumors (e.g., hepatocellular carcinoma)	Associated with a risk of rectal perforation or deep tissue injury
Cryospray nozzle	Mucosa-limited lesions (e.g., small gastrointestinal polyps)	Insufficient for LRC: Fails to achieve deep tumor ablation

### Cryotherapy classification for LRC

Cryotherapy for LRC is categorized into radical and palliative modalities, with distinct goals, eligibility criteria (28–31) (**Table 2**).

**Table 2:** Radical vs. palliative cryotherapy for LRC.

Parameter	Radical cryotherapy	Palliative cryotherapy
Primary goal	Complete tumor eradication (clinical complete response, cCR)	Tumor burden reduction; symptom relief (bleeding/obstruction) (29–31)
Eligibility criteria	• High/moderately differentiated adenocarcinoma (G1–G2) • Tumor upper pole ≤5 cm from anal verge • Tumor diameter ≤3 cm • cT0–2N0M0 • No prior therapy	• Pathologically confirmed LRC (any differentiation, including G3) • Tumor upper pole ≤5 cm from anal verge • cT3–4N+M+ or residual/recurrent tumor • Symptom burden (bleeding ≥grade 2 or pain VAS ≥4)

### Cryotherapy indications and contraindications

#### Radical cryotherapy eligibility [Based on updated guidelines and institutional criteria]

1. Histologically confirmed rectal adenocarcinoma (high/moderate differentiation, G1–G2) via endoscopic biopsy (≥2 samples per case) (32–34).
2. Tumor location: Upper pole ≤5 cm from anal verge (measured via rigid proctoscopy) (32–34).
3. Tumor size: Maximum diameter ≤3 cm (assessed via pelvic MRI + endorectal ultrasound) (32–34).
4. Clinical stage: cT_0–2_N_0_M_0_.
5. Patient factors: No prior neoadjuvant therapy; explicit preference for sphincter preservation.

#### Palliative cryotherapy eligibility

1. Pathologically confirmed LRC (any differentiation, including signet-ring cell carcinoma).
2. Tumor upper pole ≤5 cm from anal verge (rigid proctoscopy).
3. Clinical stage: cAnyTN+ or M+; or cT0–2N0M0 with poor differentiation (G3); or residual/recurrent tumor post-radiotherapy/chemotherapy.

#### Contraindications

1. Hematologic disorders: Coagulation dysfunction (INR >1.5, platelets <50 ×10^9^/L) or active bleeding diathesis.
2. Anticoagulant use: Inability to discontinue warfarin/heparin etc. For ≥7 days (no bridging possible).
3. Malignant cachexia.

All patients undergo multidisciplinary team (MDT) discussion involving colorectal surgeons, radiation oncologists, medical oncologists, radiologists, radiation physicist, endoscopist, and pathologists to balance oncologic goals and functional preservation (35, 36).

### Pre-cryoablation preparation and procedure

#### Pre-procedure assessment (standardized protocol)

1. Tumor characterization:

A. Histology: Endoscopic biopsy to confirm differentiation grade (all cases reviewed by two independent pathologists).
B. Imaging: Pelvic MRI (high-resolution T2-weighted imaging) for tumor depth and mesorectal lymph nodes; endorectal ultrasound for T-stage refinement; CT chest/abdomen to rule out distant metastasis.
C. Functional assessment: Wexner anal function score (Wexner scores range from 0 [perfect continence] to 20 [complete incontinence]).

2. Patient preparation:

A. Bowel preparation: Polyethylene glycol (4 L) administered 24 hours prior to the procedure.
B. Antibiotic prophylaxis: Administered 30 minutes before the procedure.

#### Anesthesia

• Preferred: Sacral canal anesthesia (1.5% Lidocaine, 15 mg) for sphincter relaxation and pain control.
• Alternative: Perineal nerve block (2% lidocaine, 10 mL) for patients with spinal anesthesia contraindications.
• General anesthesia: Only used in cases with abnormal spinal anatomy.

#### Procedural steps (standardized protocol)

**Figure 1:**
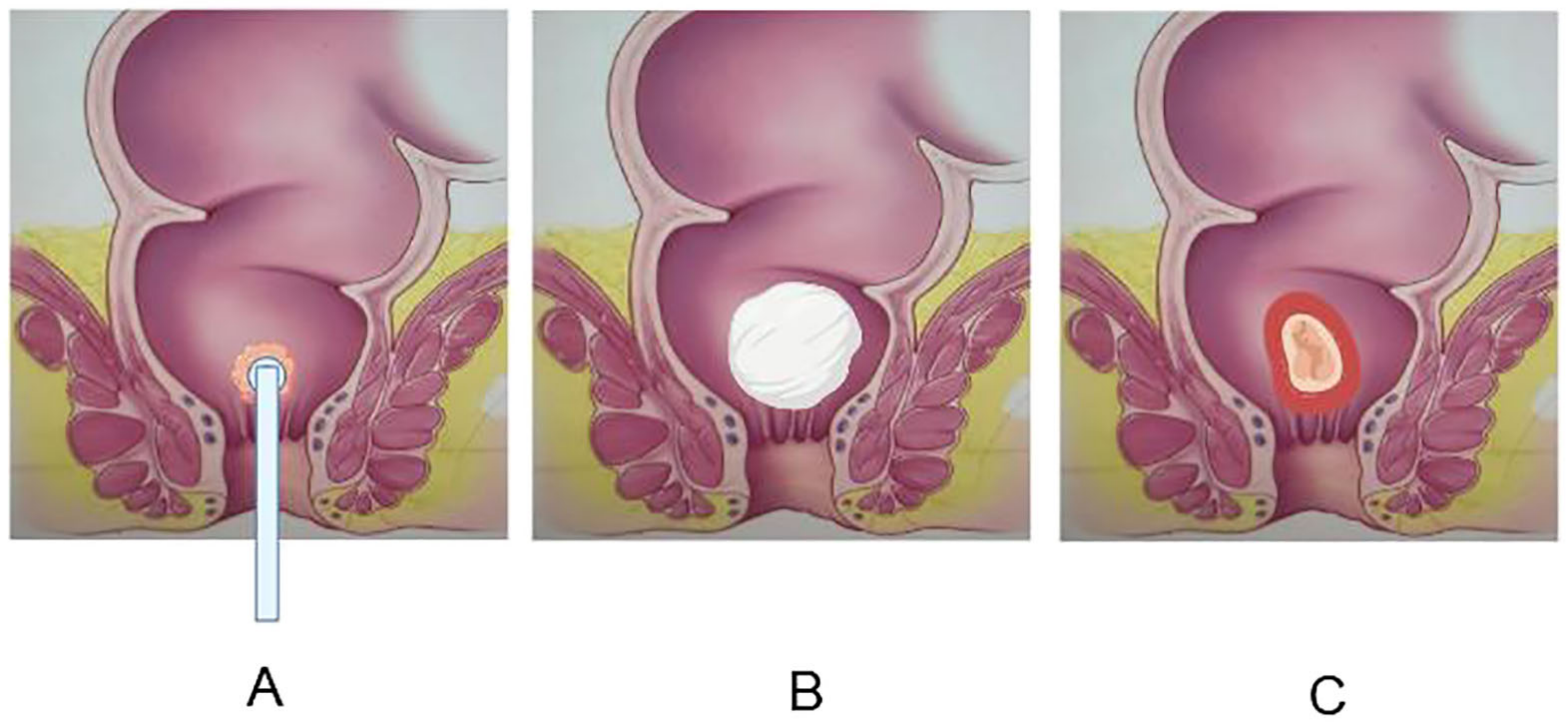
Schematic of cryotherapy. **(A)** Ice ball applied to the tumor surface. **(B)** Tumor fully covered by the ice ball. **(C)** Post-procedure tumor disappearance and ulcer formation.

**Figure 2:**
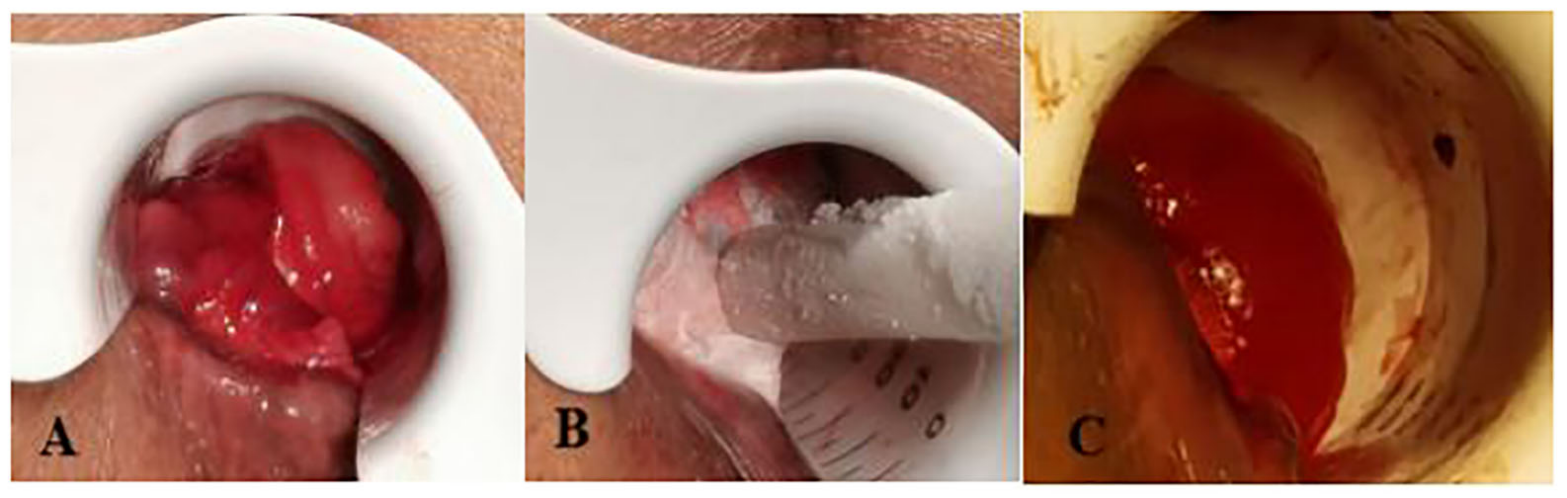
Intraoperative steps of radical cryotherapy (56-year-old male, cT2N0M0 LRC; tumor upper pole 2 cm from anus with no recurrence at 2 years of follow-up; the Wexner score was 4 indicating good functional outcome). **(A)** Preoperative proctoscopic view showing a exophytic, erythematous tumor on the anterior rectal wall. **(B)**Ice ball formation: Ice ball fully encompassing the tumor. **(C)** Post-thaw inspection: The ablation site demonstrates dark discoloration and edema, indicative of coagulative necrosis.

1. The patient is positioned in the lithotomy position (hips flexed at 90°) to optimize tumor exposure.
2. Inserted a rigid proctoscope to visualize the tumor, and cleaned the lesion surface with normal saline to remove debris.
3. The pie-shaped probe (1.5 cm × 1.5 cm) is placed directly on the tumor surface (**Figures 1A**, **2A**) and secured with gentle pressure to ensure adequate contact.
4. Initiate the freezing cycle: The target temperature is set to -40 °C (APCA1–3 system) and maintained until the visualized ice ball fully encompasses the tumor with a minimum 5-mm margin beyond its borders (confirmed via intraoperative ultrasound; **Figures 1B**, **2B**).
5. Thawing phase: Deactivate the probe; allow natural thawing until ice ball completely resolves (**Figure 2C**).
6. Repeat freeze-thaw cycle once.
7. Inspect the ablation site: Check for active bleeding; suture (3–0 Vicryl) any visible vessels.

#### Post-cryoablation management

1. In-Hospital Observation

• Monitoring: Vital signs; abdominal tenderness; hematochezia and urine output.
• Prophylaxis:
  ○ Antibiotics for 24–48 hours.
  ○ Laxatives to prevent constipation-related bleeding.

• Symptom control: Postprocedure pain is typically mild and adequately controlled with conventional oral gesics.

2. Follow-Up and Monitoring (Standardized Protocol in our center) (**Table 3**)

**Table 3:** Follow-up and monitoring strategies.

Time point	Assessments
1 week	Digital rectal exam; symptom assessment (pain, bleeding); Wexner score
3 months	Colonoscopy (to confirm cCR in radical cases); pelvic MRI; CEA
6 months	DRE; pelvic MRI; CEA; Wexner score
1–2 years	Digital rectal exam (DRE) every 3 months; pelvic MRI every 3 months; carcinoembryonic antigen (CEA) every 3 months; colonoscopy every 6 months
3–5 years	DRE (q6m); pelvic MRI (q6m); CEA (q6m); colonoscopy (q12m)

#### Possible complications and mitigation strategies

1. Major Bleeding (Grade ≥3)

• Presentation: Hematochezia with clots (≥50 mL/24h); no hypotension (systolic >90 mmHg).
• Etiology: Vascular exposure at the necrotic ulcer site (confirmed via colonoscopy) (**Figure 1C**).
• Mitigation Strategies:
  ○ Intraoperative: Ultrasound-guided identification of submucosal vessels; suture ligation of suspicious vessels.
  ○ Postoperative: Avoidance of nonsteroidal anti-inflammatory drugs (NSAIDs); continued use of laxatives.

2. Rectal Stricture (Grade 2–3)

• Presentation: Dyschezia; partial obstruction.
• Etiology: Excessive scar tissue.
• Mitigation:
  ○ Prevention: avoid ablation of >50% of rectal circumference.
  ○ Treatment: Digital dilation (weekly, for 4–6 weeks) for mild strictures; endoscopic balloon dilation for severe strictures.

3. Retroperitoneal Infection (Grade 3)

No cases reported; mitigation strategies based on literature: Prophylactic antibiotics; CT-guided drainage for abscesses; IV antibiotics may be useful if infection occurs (37).

Additional pitfalls include inaccurate staging of tumor depth or lymph node involvement, which could lead to inappropriate patient selection. To minimize this, all patients undergo high−resolution pelvic MRI and endorectal ultrasound reviewed by dedicated radiologists. For anterior wall tumors, proximity to the vagina or prostate requires real−time transperineal ultrasound monitoring to avoid genitourinary injury.

## Cryoablation in the context of other organ-preserving modalities

Several organ-preserving alternatives exist for patients with low rectal cancer who are unfit for or decline radical surgery. Contact X-ray brachytherapy (CXB) delivers a high radiation dose to superficial tumors (≤3 cm) and has reported local control rates of approximately 90% in selected series, but it requires specialized equipment and expertise (38–40). Interstitial high-dose-rate brachytherapy (HDR-BT) can treat slightly larger lesions and offers favorable toxicity, yet it necessitates multiple applicator insertions and careful dosimetry (41). External beam radiotherapy (EBRT), often combined with chemotherapy, can achieve clinical complete response in 20–40% of patients but is associated with acute and late toxicities (42). Compared to these radiation-based approaches, cryoablation offers distinct advantages: directly resulting in tumor elimination, real-time ultrasound monitoring of the ice ball margin, a rapid learning curve for colorectal surgeons, low equipment cost, and potential immunomodulatory effects (e.g., abscopal phenomenon). However, it is currently indicated only for tumors within 5 cm of the anal verge and ≤3 cm in diameter. The choice of modality should be individualized through multidisciplinary discussion, taking into account tumor characteristics, patient preferences, and institutional resources.

## Summary of institutional experience and outcomes

Over the past five years, we have applied this protocol in over 100 selected patients with LRC. Data from our institutional experience are presented as simple counts or percentages to illustrate the applicability of the perspective. In this cohort, approximately 80% were treated with radical intent and 20% with palliative intent. Among those treated radically, a clinical complete response (cCR)was observed in the vast majority at 3 months, and the rate of major complication (Grade ≥3) was below 3%. Functional outcomes were excellent, with a median Wexner score of 1 at 6 months. These preliminary data support the safety and efficacy of the protocol, but formal analysis of long-term oncologic outcomes is ongoing and will be reported in future publications.

We acknowledge that this protocol reflects the experience of a single, high-volume center. Further validation through multiinstitutional collaborations and prospective registries is warranted to generalize these findings.

In our experience, cryoablation provides three key advantages over other transanal local treatments such as Transanal endoscopic microsurgery: first, the controllable ice ball enables precise and complete tumor coverage, particularly in the critical dimension perpendicular to the intestinal wall; second, its operational repeatability enhances the oncologic result; third, technical proficiency for cryoablation can be acquired more rapidly than for procedures like TEM, which demands mastery of complex endoscopic skills (43). This accessibility may allow for its successful implementation by a broader range of colorectal surgeons, not only those in high-volume tertiary referral centers. To further establish this technique, the following steps are imperative:

1. Preoperative staging accuracy: the critical role of precise staging was reaffirmed; the combination of pelvic MRI and endorectal ultrasound was essential in minimizing staging errors and proper patient selection.
2. Combined therapy potential: neoadjuvant radiotherapy followed by cryoablation for locally advanced disease to preserve sphincters.
3. Multicenter prospective trials: Validating the efficacy and safety across diverse patient populations through multicenter prospective trials.

Meanwhile, some patients with LRC and distant metastases still chose cryoablation as an alternative treatment because of their preference in sphincter-preservation. Notably, in a subset of these patients, we observed regression of metastatic lesions following cryoablation —a finding consistent with the “abscopal effect.” Concomitantly, we detected a post-treatment increase in peripheral blood CD4^+^ and CD8^+^ T cell counts. Hence, we are conducting investigations to further characterize this immunomodulatory effect and its association with the abscopal response to evaluate the immune effect of cryoablation.

In conclusion, cryoablation serves as a minimally invasive and sphincter-preserving therapeutic strategy for a defined population with LRC. Its key role in sphincter preservation makes it become an attractive approach for patients with preference in avoiding colostomata, particularly the elderly patients or those with concurrent medical comorbidity.
